# Analysis of virulence phenotypes and antibiotic resistance in clinical strains of *Acinetobacter baumannii* isolated in Nashville, Tennessee

**DOI:** 10.1186/s12866-020-02082-1

**Published:** 2021-01-09

**Authors:** Ranashia L. Boone, Briana Whitehead, Tyra M. Avery, Jacky Lu, Jamisha D. Francis, Miriam A. Guevara, Rebecca E. Moore, Schuyler A. Chambers, Ryan S. Doster, Shannon D. Manning, Steven D. Townsend, Leon Dent, Dana Marshall, Jennifer A. Gaddy, Steven M. Damo

**Affiliations:** 1 Department of Life and Physical Sciences, Fisk University, Talley-Brady Hall, 1000 17th Ave. N, Nashville, TN 37208 USA; 2 Department of Pathology, Microbiology, and Immunology, Vanderbilt University School of Medicine, Nashville, TN USA; 3 Department of Chemistry, Vanderbilt University, Nashville, TN USA; 4 Department of Medicine, Division of Infectious Diseases, Vanderbilt University Medical Center, A2200 Medical Center North, 1161 21st Avenue South, Nashville, TN 37232 USA; 5 Department of Microbiology and Molecular Genetics, Michigan State University, East Lansing, MI USA; 6 Department of Pathology, Anatomy, and Physiology, Meharry Medical College, Nashville, TN USA; 7 Trauma Services, Phoebe Putney Memorial Hospital, Albany, GA USA; 8 Department of Veterans Affairs, Tennessee Valley Healthcare Systems, Nashville, TN USA; 9 Department of Biochemistry, Vanderbilt University, Nashville, TN USA; 10 Center for Structural Biology, Vanderbilt University, Nashville, TN USA

**Keywords:** Biofilm, Motility, *Acinetobacter baumannii*, Antimicrobial resistance, Antibiotics

## Abstract

**Background:**

*Acinetobacter baumannii* is a gram-negative bacterium which causes opportunistic infections in immunocompromised hosts. Genome plasticity has given rise to a wide range of strain variation with respect to antimicrobial resistance profiles and expression of virulence factors which lead to altered phenotypes associated with pathogenesis. The purpose of this study was to analyze clinical strains of *A. baumannii* for phenotypic variation that might correlate with virulence phenotypes, antimicrobial resistance patterns, or strain isolation source. We hypothesized that individual strain virulence phenotypes might be associated with anatomical site of isolation or alterations in susceptibility to antimicrobial interventions.

**Methodology:**

A cohort of 17 clinical isolates of *A. baumannii* isolated from diverse anatomical sites were evaluated to ascertain phenotypic patterns including biofilm formation, hemolysis, motility, and antimicrobial resistance. Antibiotic susceptibility/resistance to ampicillin-sulbactam, amikacin, ceftriaxone, ceftazidime, cefotaxime, ciprofloxacin, cefepime, gentamicin, levofloxacin, meropenem, piperacillin, trimethoprim-sulfamethoxazole, ticarcillin- K clavulanate, tetracyclin, and tobramycin was determined.

**Results:**

Antibiotic resistance was prevalent in many strains including resistance to ampicillin-sulbactam, amikacin, ceftriaxone, ceftazidime, cefotaxime, ciprofloxacin, cefepime, gentamicin, levofloxacin, meropenem, piperacillin, trimethoprim-sulfamethoxazole, ticarcillin- K clavulanate, tetracyclin, and tobramycin. All strains tested induced hemolysis on agar plate detection assays. Wound-isolated strains of *A. baumannii* exhibited higher motility than strains isolated from blood, urine or Foley catheter, or sputum/bronchial wash. *A. baumannii* strains isolated from patient blood samples formed significantly more biofilm than isolates from wounds, sputum or bronchial wash samples. An inverse relationship between motility and biofilm formation was observed in the cohort of 17 clinical isolates of *A. baumannii* tested in this study. Motility was also inversely correlated with induction of hemolysis. An inverse correlation was observed between hemolysis and resistance to ticarcillin-k clavulanate, meropenem, and piperacillin. An inverse correlation was also observed between motility and resistance to ampicillin-sulbactam, ceftriaxone, ceftoxamine, ceftazidime, ciprofloxacin, or levofloxacin.

**Conclusions:**

Strain dependent variations in biofilm and motility are associated with anatomical site of isolation. Biofilm and hemolysis production both have an inverse association with motility in the cohort of strains utilized in this study, and motility and hemolysis were inversely correlated with resistance to numerous antibiotics.

**Supplementary Information:**

The online version contains supplementary material available at 10.1186/s12866-020-02082-1.

## Introduction

*Acinetobacter baumannii* is a multi-drug resistant gram-negative bacterial pathogen that causes severe infections in compromised human patients and is a global health threat [1]. While 25 species of *Acinetobacter* have been discovered using DNA–DNA hybridization, 80% of clinical infections caused by *Acinetobacter* can be attributed to the *A. calcoaceticus – A. baumannii* complex, a group of bacteria comprised of nonfermenting, aerobic, Gram-negative coccobacilli which can be identified by colony morphology, Gram staining, growth at 37 °C, a negative oxidase test result, and oxidation of glucose [2]. A large proportion of these infections are nosocomially acquired, particularly within intensive care units where patients are immunosuppressed [3]. *A. baumannii* causes a wide range of infections in diverse anatomical sites including urinary tract infections, sepsis, pneumonia, as well as skin and soft-tissue infections. One of the most common disease manifestations associated with this pathogen is ventilator acquired pneumonia (VAP) [3], although cases of community acquired pneumonia have also been reported [4]. The mortality rate correlated with VAP is as high as 25% in intubated patients and surpasses 50% in vulnerable ICU patients requiring vasopressors [3].

The prevalence of multi- and pan-drug resistant strains of *A. baumannii* has been increasing, confounding clinicians’ ability to effectively treat these infections [5]. Recent studies have shown that *A. baumannii* exploits a repertoire of factors and processes to promote antibiotic resistance at a cellular level. One of the mechanisms by which *A. baumannii* circumnavigates antibiotic pressure is its ability to form biofilms. Biofilm structures are multicellular aggregates of microorganisms which adhere to abiotic or biotic surfaces, as well as each other, and secrete a polymeric extracellular matrix to develop a tertiary architectural structure of cells [6, 7]. This protective extracellular matrix impedes antibiotic penetrance of the cells inside of the biofilm, thereby decreasing susceptibility to antibiotic pressure [6–8]. *A. baumannii* forms tenacious biofilms on abiotic surfaces including medical devices such as catheters, implants and ventilators [6–8]. Biofilms aid bacterial survival and persistence in hospital settings for extended periods of time, leading to multiple outbreaks in health care facilities [2].

Bacterial pathogens such as *A. baumannii* facilitate physiological processes like biofilm formation, motility, and virulence regulation through a network of signaling cascades and environmental sensing mechanisms [8]. One pathway which governs biofilm, motility, and virulence is called quorum sensing [9]. Quorum sensing is the ability of bacterial cells to communicate and respond to bacterial cell population by releasing small diffusible signal molecules known as autoinducers [9]. *A. baumannii* and other bacteria produce autoinducers called acyl homoserine lactones (AHLs), which have been implicated in bacterial quorum sensing. AHL production by bacteria promotes the induction of virulence factors, motility, plasmid transfer, and biofilm formation [10]. A reduction in biofilm formation of up to 40% can be seen in *A. baumannii* that lack the *abaI* gene, which is responsible for producing AHL [7]. The ability of bacteria to operate through cell-cell communication allows advantages for survival like host colonization, the formation of biofilm, defense against competing organisms, and evolution [9].

Bacteria have developed motility features that permit movement across solid surfaces and aqueous environments [11]. Mechanisms of bacterial motility are distinct and are influenced by the environment and structure of each bacterium. The main types of motility include: swimming, swarming, gliding, and twitching [11]. *A. baumannii* have a type IV pilus appendage that extends and retracts allowing for twitching motility across semisolid and abiotic surfaces [12–14]. Additionally, *A. baumannii* exhibits surface motility independent of the type IV pili through the synthesis of 1, 3-diaminopropane [15]. Motility is a critical process for pathogenesis as it promotes bacterial spread to and from specific sites of infection as well as evasion of the host immune system [11–15].

Another important pathogenesis pathway is hemolysis, or the process by which red blood cells are lysed [16]. Bacterial pathogens have evolved the ability to induce hemolysis in order to extract nutrients, such as iron, from host cells. Previous studies in *Acinetobacter* have identified all three types of hemolytic activity; β-hemolysis being the most common in this genus [17]. Within the vertebrate host, micronutrients such as iron are bound to host molecules such as heme, hemoglobin, transferrin, lactoferrin, and ferritin to limit growth and proliferation of invading microorganisms which require these micronutrients as cofactors for a variety of cellular processes.

Here, we investigate bacterial strain phenotypes including hemolysis, motility, biofilm formation and antimicrobial resistance of 17 clinical isolates that were isolated from diverse anatomical sites including blood, urine and Foley catheter, bronchial wash, sputum, abdominal cavity, and wound sites from a cohort of patients in Nashville, Tennessee. Biofilm formation and motility was explored independently along with systematic correlation between the two, while the relationship between anatomical site of isolation of each strain, hemolysis, antibiotic resistance, motility, and biofilm formation was also investigated.

## Methods and methods

### Bacterial strains, antimicrobial susceptibility, and media conditions

Seventeen clinical isolates of *A. baumannii* were characterized in this study. Strains were chosen from diverse anatomical origin and from a wide range of disease presentations including urinary tract, respiratory, wound, intra-abdominal infections, and bacteremia. Strains were collected in this pilot study from January of 2010 through August of 2012. Antimicrobial susceptibility to ampicillin-sulbactam (A/S), amikacin (AK), ceftriaxone (CAX), ceftazidime (CAZ), cefotaxime (CFT), ciprofloxacin (CP), cefepime (CPE), gentamicin (GM), levofloxacin (LVX), meropenem (MER), piperacillin (PI), trimethoprim-sulfamethoxazole (T/S), tetracycline (TE), ticarcillin-K clavulanate (TIM), and tobramycin (TO) was determined at the Nashville General Hospital clinical laboratory and values of “susceptible”, “non-susceptible” or “intermediate” per International Organization for Standardization (ISO) 20776–1:2019 guidelines, was determined [18]. A bacterial strain was considered “susceptible” when it was inhibited in vitro by a concentration of drug that is associated with a high likelihood of therapeutic success. An “intermediate” designation indicated the strain had variable inhibition in vitro*,* or was inhibited by a concentration of drug that is associated with an uncertain therapeutic effect. And strains were designated resistant or “non-susceptible” to a given antibiotic when the strain was not inhibited in vitro by a concentration of drug that is associated with therapeutic success. Additionally, anatomical site source of culture were retrieved in a de-identified manner from the electronic medical record system. Approval to characterize the de-identified bacterial isolates was provided by the affiliated Meharry Medical College Institutional Review Board (IRB 081204AAH23119). Reference laboratory strains of *A. baumannii* including 17978 and the 19606 T type strain (ATCC, Manassas, Virginia) from patients with meningitis and urinary tract infection, respectively, were also evaluated for comparison. All bacterial strains were stored as glycerol stock at -80 °C until use. All isolates were grown in Luria-Bertani (LB) broth at 37 °C in room air under shaking conditions overnight at 180 rpm to an optical density of 600 nm (OD_600_) between 0.8–1.0.

### Hemolysis assay

*A.baumannii* isolates were streaked from glycerol stocks onto Tryptic Soy Agar plates containing 5% sheep blood (blood agar plates), and sub-cultured in LB broth overnight. The following day, bacteria were subjected to serial dilution (up to 10^− 6^ dilution) and 3 μL of culture was plated onto fresh blood agar plates for visualization of β-hemolysis. Plates were visually inspected for the clearing of red blood cells surrounding bacterial colonies and the underside of the plate was imaged. β-hemolysis was evaluated semi-quantitatively in a blinded fashion by two independent investigators using a scale in which “+” signified low hemolysis, “++” signified moderate hemolysis, and “+++” signified high levels of hemolysis.

### Bacterial motility analysis

To determine cell motility, swimming agar plates were used containing Luria-Bertani and 0.3% agar as previously described [12]. Swimming agar plates were inoculated in the center with 3 μL of overnight culture. Plates were then incubated at 37 °C for 24 h in dark conditions. To visualize motility of isolates, images were taken at 24 h post-inoculation and the diameter was measured to quantify migration.

### Bacterial biofilm quantification

*A. baumannii* biofilms were cultured and analyzed as previously described [19, 20]. Briefly, overnight cultures were diluted ten-fold in fresh LB broth and incubated at 37 °C for 24 h in dark stagnant conditions. The OD_600_ was measured to determine the density of the bacterial cells in each culture. Biofilm formation was assessed by crystal violent staining. Crystal violet (1%) was used to stain bacterial cells for 30 min on a shaker, decanted, and then washed twice with distilled water. To solubilize the crystal violet, 200 μL of 80%/20% ethanol/acetone solution was added to each well and absorbance at 560 nm was recorded. The biofilm formation of each isolate was normalized to its respective total mass by using ratios of absorbance at 560 nm (crystal violet staining) and 600 nm (biomass). Each biofilm assay consisted of 4 technical replicates and the assay was repeated at least three times using fresh overnight cultures. All OD_560_/OD_600_ ratios above 1.8, the median value of all strains tested, were considered strong biofilm formers, while strains exhibiting values below this were considered weak biofilm formers. To determine this cutoff value, absorbance values were subjected to the D’Agostino and Pearson test of normality as previously described [21].

### Statistical analyses

Statistical analyses of biofilm formation and motility were performed using a one-way ANOVA with either Tukey’s or Dunnett’s post hoc correction for multiple comparisons. All reported *P* values are adjusted to account for multiple comparisons. Analysis of correlations between phenotypes was performed using either Spearman’s or Pearson’s correlation analyses. *P* values of ≤0.05 were considered significant. All data analyzed in this work were derived from at least three biological replicates. Statistical analyses were performed using GraphPad Prism 6 software (GraphPad Prism Software Inc., La Jolla, California).

## Results

### Susceptibility to antibiotics

Resistance to antibiotics was widespread among the *A. baumannii* isolates tested (Table 1) and varied based on isolation site. All *A. baumannii* clinical isolates obtained from patient blood samples were multi-drug resistant (MDR), and were specifically resistant to ceftriaxone, ceftazidime, cefotaxime, ciprofloxacin, cefepime, gentamicin, levofloxacin, meropenem, piperacillin, trimethoprim-sulfamethoxazole, ticarcillin- K clavulanate, and tobramycin. All *A. baumannii* strains isolated from urine or Foley catheter sources were MDR and were specifically resistant to ceftriaxone, ceftazidime, cefotaxime, ciprofloxacin, levofloxacin, meropenem, piperacillin, trimethoprim-sulfamethoxazole, ticarcillan- K clavulanate. Five out of seven sputum isolates were resistant to ceftriaxone, ceftazidime, cefotaxime, ciprofloxacin, gentamicin, and T/S. Three out of four wound isolates were susceptible to ampicillin-sulbactam, amikacin, ceftazidime, ciprofloxacin, levofloxacin, trimethoprim-sulfamethoxazole, tetracycline, tobramycin. Four out of seven sputum isolates were sensitive to ampicillin-sulbactam, amikacin, and four out of seven sputum isolates were resistant to cefepime, levofloxacin. And, interestingly, the single isolate derived from the abdominal cavity of a patient was susceptible to all antibiotics tested.

**Table 1:**
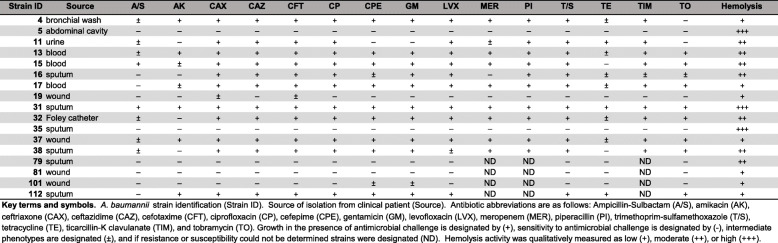
*A. baumannii* clinical strains, isolation source, antibiotic susceptibility, and hemolysis profiles

### Hemolysis

All strains tested in this cohort showed at least low levels of hemolysis when qualitatively analyzed on agar plates containing 5% sheep blood (Supplemental Fig. 1 and Table 1). All strains isolated from wounds exhibited low levels of hemolysis; however, seven strains isolated from urine, blood and sputum showed moderate hemolysis. Interestingly, two out of three strains with the highest hemolysis (*A. baumannii* strains 5 and 35, isolated from the abdominal cavity and sputum, respectively) were also susceptible to all antibiotics. It is pertinent to note that two strains, one from sputum and a wound isolate, had moderate and low hemolysis, respectively, were also sensitive to all antibiotics that they were tested against, but sensitivity to three different antibiotics could not be determined in these strains.

### Motility

**Fig. 1:**
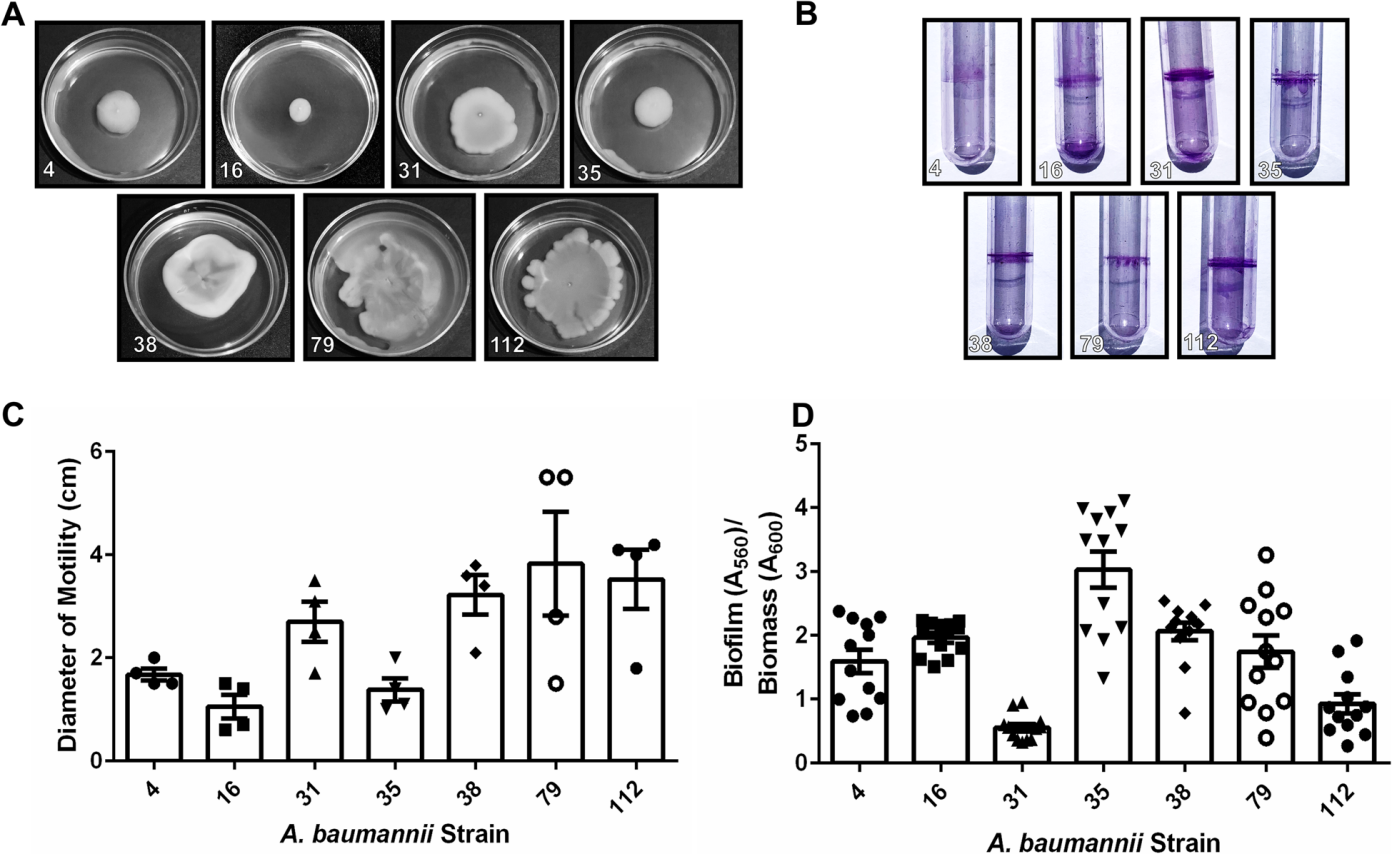
Analysis of motility and biofilm phenotypes from *A. baumannii* strains isolated from sputum or bronchial wash samples. **a** Motility agar plates 24 h post-inoculation with *A. baumannii* clinical isolate strains. **b** Crystal violet stained *A. baumannii* biofilms in polystyrene tubes. **c** Quantitative analysis of bacterial motility as determined by measurement of diameter of bacterial cells present on the plate. Bars indicate mean values (+/− standard error mean error bars) with individual biological replicates indicated by discrete points (*n*=4). **d** Quantitative analysis of ratio of biofilm to biomass. Biofilm was determined by solubilization of crystal violet and spectrophotometric measurement at OD_560_. Biomass was determined by spectrophotometric measurement at OD_600_. Bars indicate mean values (+/− standard error mean error bars) with individual data points indicated by discrete points (*n*=3 biological replicates with 3–4 technical replicates per experiment)

**Fig. 2:**
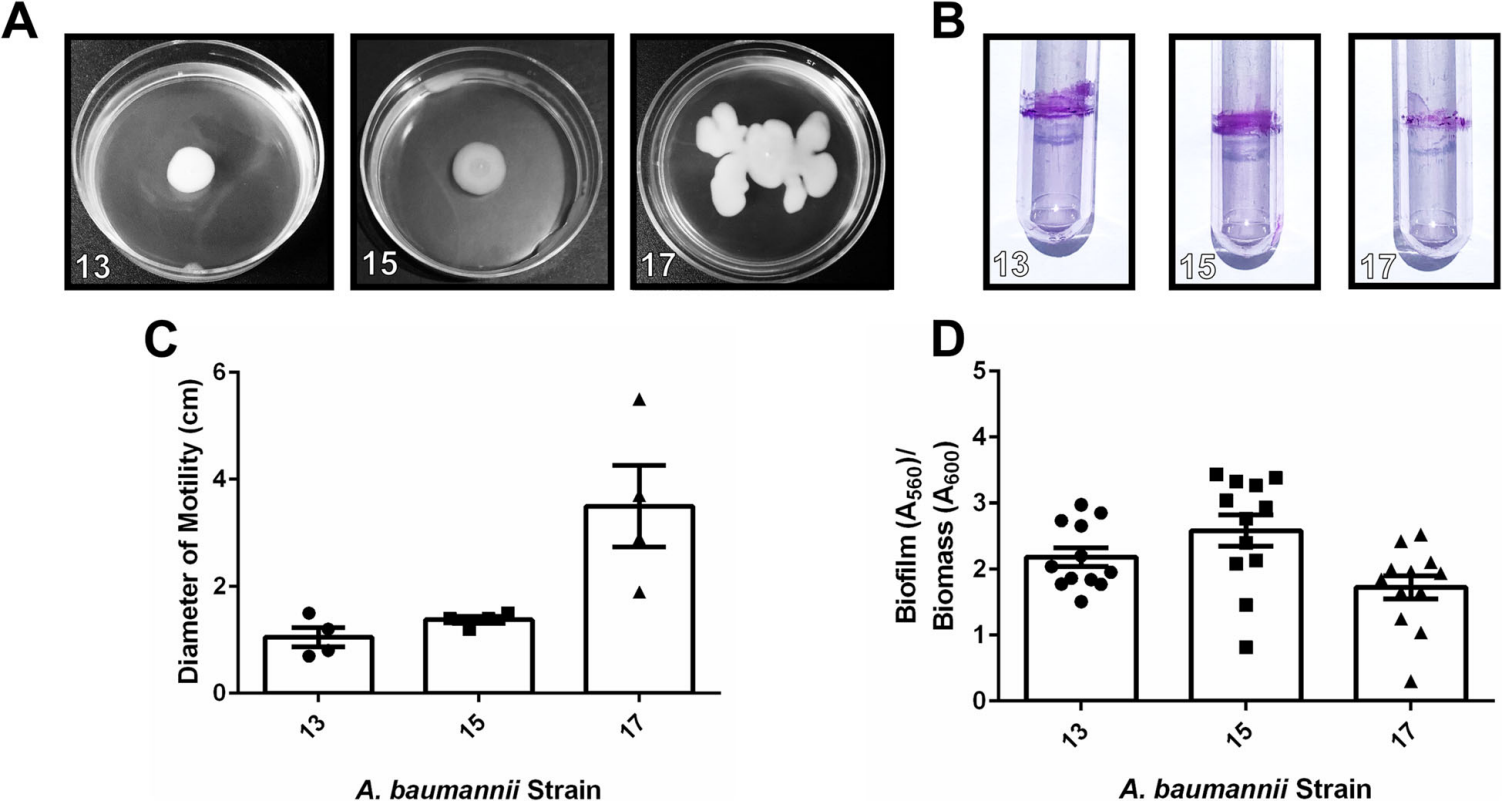
Analysis of motility and biofilm phenotypes from *A. baumannii* strains isolated from blood samples. **a** Motility agar plates 24 h post-inoculation with *A. baumannii* clinical isolate strains. **b** Crystal violet stained *A. baumannii* biofilms in polystyrene tubes. **c** Quantitative analysis of bacterial motility as determined by measurement of diameter of bacterial cells present on the plate. Bars indicate mean values (+/− standard error mean error bars) with individual biological replicates indicated by discrete points (*n=*4). **d** Quantitative analysis of ratio of biofilm to biomass. Biofilm was determined by solubilization of crystal violet and spectrophotometric measurement at OD_560_. Biomass was determined by spectrophotometric measurement at OD_600_. Bars indicate mean values (+/− standard error mean error bars) with individual data points indicated by discrete points (*n=*3 biological replicates with 3–4 technical replicates per experiment)

**Fig. 3:**
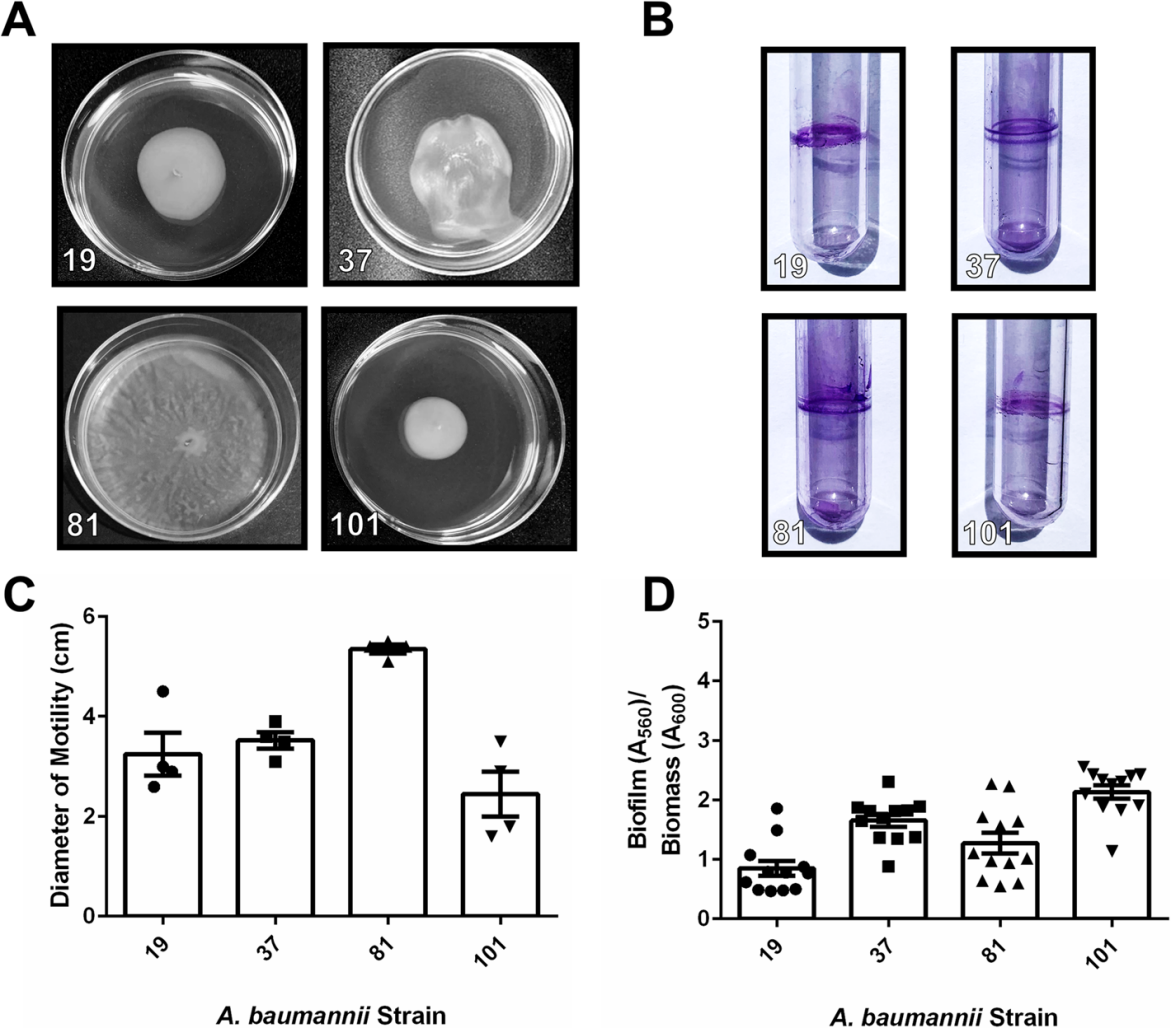
Analysis of motility and biofilm phenotypes from *A. baumannii* strains isolated from wound samples. **a** Motility agar plates 24 h post-inoculation with *A. baumannii* clinical isolate strains. **b** Crystal violet stained *A. baumannii* biofilms in polystyrene tubes. **c** Quantitative analysis of bacterial motility as determined by measurement of diameter of bacterial cells present on the plate. Bars indicate mean values (+/− standard error mean error bars) with individual biological replicates indicated by discrete points (*n=*4). **d** Quantitative analysis of ratio of biofilm to biomass. Biofilm was determined by solubilization of crystal violet and spectrophotometric measurement at OD_560_. Biomass was determined by spectrophotometric measurement at OD_600_. Bars indicate mean values (+/− standard error mean error bars) with individual data points indicated by discrete points (*n=*3 biological replicates with 3–4 technical replicates per experiment)

**Fig. 4:**
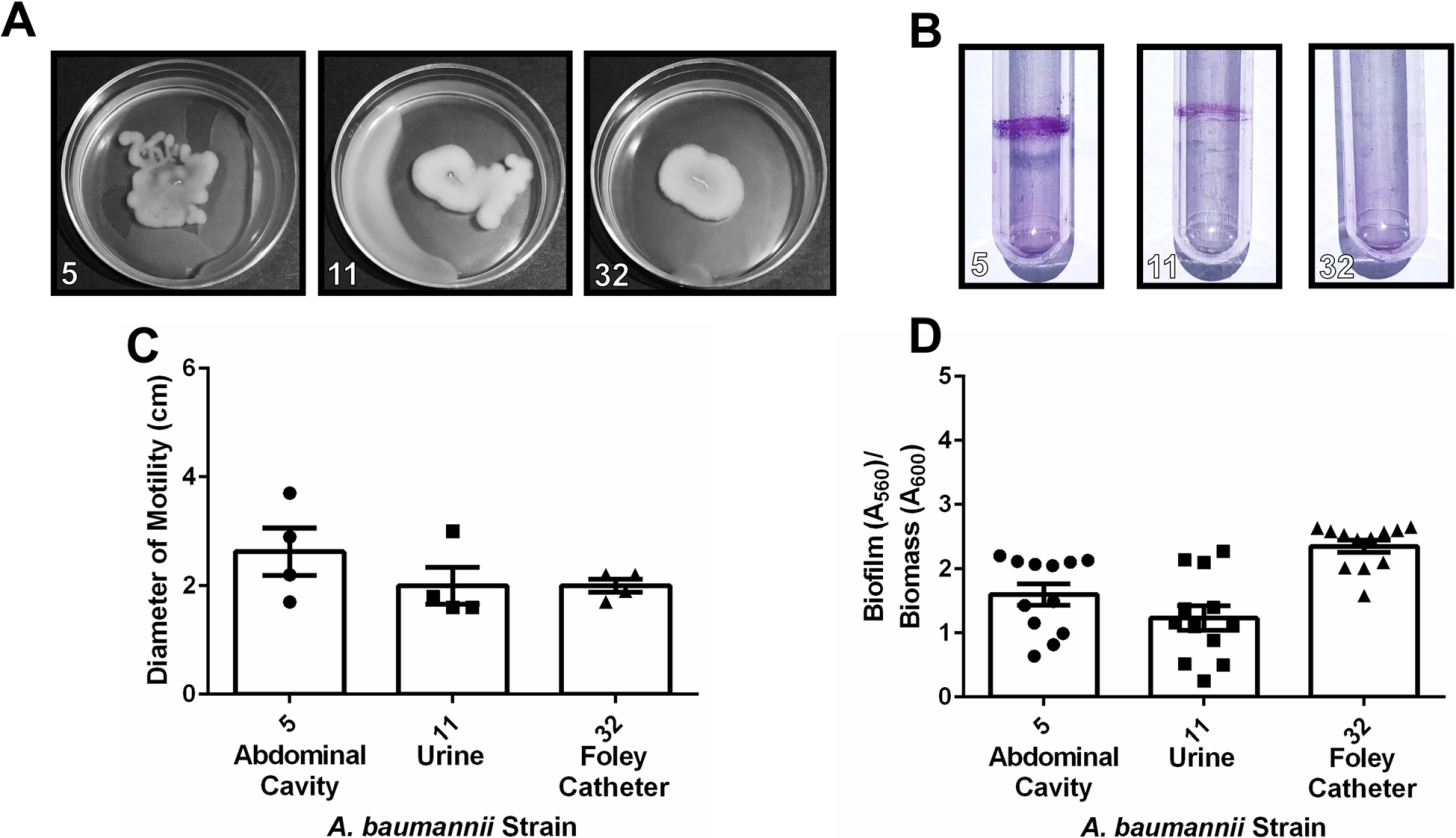
Analysis of motility and biofilm phenotypes from *A. baumannii* strains isolated from abdominal cavity, urine, and Foley catheter samples. **a** Motility agar plates 24 h post-inoculation with *A. baumannii* clinical isolate strains. **b** Crystal violet stained *A. baumannii* biofilms in polystyrene tubes. **c** Quantitative analysis of bacterial motility as determined by measurement of diameter of bacterial cells present on the plate. Bars indicate mean values (+/− standard error mean error bars) with individual biological replicates indicated by discrete points (*n=*4). **d** Quantitative analysis of ratio of biofilm to biomass. Biofilm was determined by solubilization of crystal violet and spectrophotometric measurement at OD_560_. Biomass was determined by spectrophotometric measurement at OD_600_. Bars indicate mean values (+/− standard error mean error bars) with individual data points indicated by discrete points (*n=*3 biological replicates with 3–4 technical replicates per experiment)

Analysis of motility of all clinical *A. baumannii* strains in this cohort revealed that motility varied widely across strains, an observation consistent with laboratory strains including the type strain, *A. baumannii* 19,606 T, which has very low motility (mean motility diameter of 1.1 cm), and *A. baumannii* 17,978, which exhibits higher motility (mean motility diameter of 4.35 cm) (Supplemental Fig. 2). Strains isolated from sputum and bronchial wash had motility ranging from mean values of 1.05–3.825 cm (Fig. 1a+c), while strains isolated from blood had motility ranging from mean values of 0.7–3.5 cm (Fig. 2a+c). Strains isolated from wounds had motility ranging from mean values of 2.45–5.1 cm (Fig. 3a+c). Strains isolated from urine or Foley catheters had motility ranging from mean values of 1.7–2.0 cm (Fig. 4a+c), and the single strain (*A. baumannii* strain 5) isolated from the abdominal cavity exhibited a mean motility of 2.625 cm (Fig. 4a+c). Wound isolates were the most motile group, and were significantly more motile than isolates from sputum, bronchial wash, urine/catheter, or blood (*P*< 0.05, One-Way ANOVA).

### Biofilm

Analysis of biofilm formation by the cohort of *A. baumannii* strains isolated in Nashville, Tennessee, revealed that numerous clinical strains formed strong biofilms on polystyrene, a phenotype that is consistent with laboratory strains including the type strain, *A. baumannii* 19,606 T and *A. baumannii* 17,978 which both have capacity to form biofilms on polystyrene (Supplemental Fig. 2B+D). Strains isolated from sputum and bronchial wash had biofilm ratios ranging from mean values of 0.32–3.03 (Fig. 1). Strains isolated from blood had biofilm to biomass ratios ranging from mean values of 1.73 to 2.59 (Fig. 2b+d). Strains isolated from wounds had biofilm to biomass ratios ranging from mean values of 0.468 to 2.14 (Fig. 3b+d). Strains isolated from urine or Foley catheters had biofilm to biomass ratios ranging from mean values of 1.23 to 1.58 (Fig. 4b+d), and the single strain (*A. baumannii* strain 5) isolated from the abdominal cavity exhibited a mean biofilm to biomass ratio of 1.60 (Fig. 4b+d). Blood isolates formed the most biofilm, and were significantly higher biofilm-formers than isolates from the sputum, bronchial wash, or wounds (*P*< 0.05, One-Way ANOVA). Interestingly, 50% of the weak biofilm formers were MDR, while 71.4% of the strong biofilm formers were MDR.

### Correlation analyses

**Fig. 5:**
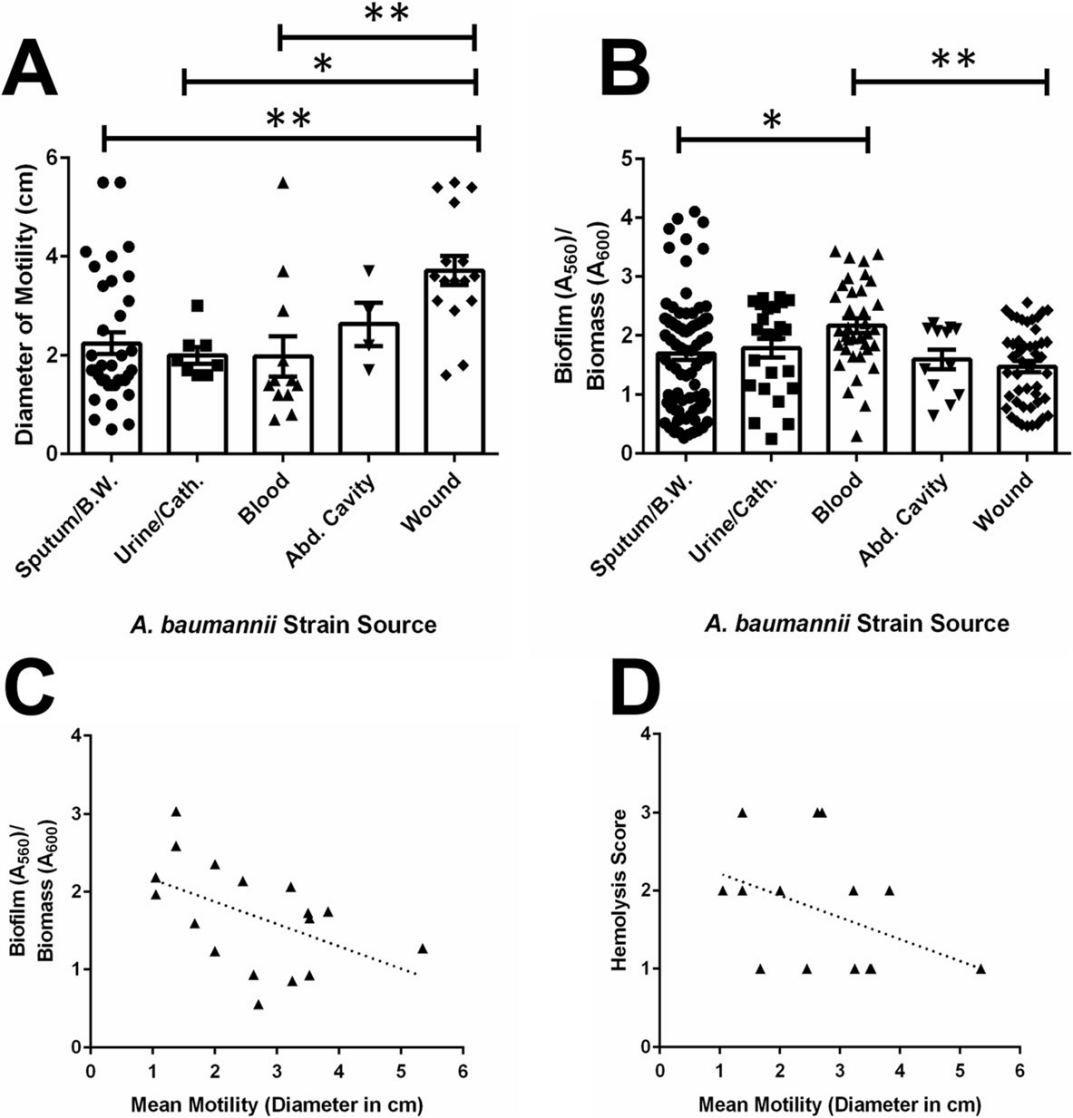
Correlation analyses of *A. baumannii* virulence phenotypes. **a** Analysis of motility phenotypes from *A. baumannii* strains isolated from various sources reveals *A. baumannii* isolated from wounds has significantly higher motility than those isolated from sputum, bronchial wash, blood (*P*< 0.01, One-Way ANOVA) or urine and Foley catheter (*P*< 0.05, One-Way ANOVA). Bars indicate mean values of all strains isolated from the source (+/− standard error mean error bars) with individual measurements for each strain indicated by discrete points (*n=*4 biological replicates). **b** Analysis of biofilm formation on polystyrene by *A. baumannii* strains isolated from various sources reveals *A. baumannii* strains isolated from blood have significantly higher biofilm to biomass ratio than those isolated from sputum or bronchial wash (P< 0.05, One-Way ANOVA) or wounds (P< 0.01, One-Way ANOVA). Bars indicate mean values of all strains isolated from the source (+/− standard error mean error bars) with individual measurements for each strain indicated by discrete points (*n=*4 biological replicates). **c** Both Spearman’s and Pearson’s correlation analyses reveal an inverse relationship between biofilm formation and motility (Pearson’s R2= 0.2480 with a 95% confidence interval of − 0.7896 to − 0.02284, *P*=0.0419, Spearman’s *R=* − 0.5270 with a 95% confidence interval of − 0.8094 to − 0.04668, *P*=0.0153). **d** Spearman’s correlation analysis reveals an inverse relationship between motility and hemolysis (Spearman’s *R=*-0.4496 with a 95% confidence interval of − 0.7713 to 0.05499, *P=*0.0153)

Stratification of strains based on source of isolation (Fig. 5) revealed that strains isolated from wounds displayed the highest motility (as determined by measuring the diameter of movement across motility agar plates, Fig. 5a). Wound-isolated strains were significantly more motile than strains isolated from blood (*P*< 0.01), urine or Foley catheter (*P*< 0.05), or sputum/bronchial wash (P< 0.01), as determined by one-way ANOVA with a Tukey’s post hoc multiple comparisons test. Analysis of biofilm formation by this cohort of clinical *A. baumannii* strains revealed that isolates derived from patient blood samples formed the highest amount of biofilm to biomass (Fig. 5b). Blood isolates exhibited a mean biofilm to biomass ratio of 2.17, and formed significantly more biofilm than isolates from sputum or a bronchial wash, which had a mean biofilm to biomass ratio of 1.64 (P< 0.05, One way ANOVA) and isolates from wound samples, which had a mean biofilm to biomass ratio of 1.48 (P< 0.01, One way ANOVA). Interestingly, isolates from anatomic sites with highest motility were concomitantly associated with strains forming lower amounts of biofilm. To interrogate this further, we performed Pearson’s correlation analyses of the 17 clinical isolates to determine the relationship between motility and biofilm. The results indicated R_2_= 0.2480 with a 95% confidence interval of − 0.7896 to − 0.02284, *P*=0.0419 (Fig. 5c). Linear regression analysis of best-fit values indicates 1/slope = − 3.479. Spearman’s correlation analyses indicated *R=*-0.5270 with a confidence interval of − 0.8094 to − 0.04668, *P*=0.0153, supporting an inverse relationship between motility and biofilm formation in our sample of 17 clinical isolates. Motility was also inversely correlated with induction of hemolysis (Fig. 5d) as determined by Spearman’s correlation analysis (*R=*-0.4496 with a 95% confidence interval of − 0.7713 to 0.05499, *P=*0.0153).

Correlation analyses between *A. baumannii* strain hemolysis score and resistance to antibiotics was also performed (Supplemental Fig. 3). Spearman’s test of correlation between *A. baumannii* strain induction of hemolysis and resistance to ampicillin-sulbactam indicated *R=*0.2604 with a 95% confidence interval (CI) of − 0.2662 to 0.6673, *P*=0.1527. Spearman’s correlation of hemolysis with resistance to amikacin was *R=*-0.2259 with CI of − 0.6464 to 0.3000, *P*=0.0886, with resistance to ceftriaxone or ceftoxamine was *R=*-0.07559 with CI of − 0.5477 to 0.4330, *P*=0.0868, with resistance to ceftazidime or ciprofloxacin was *R=*-0.02713 with CI of − 0.5127 to 0.4716, *P*=0.0769, with resistance to cefepime was *R=*-0.1751 with CI of − 0.6146 to 0.3473, *P*=0.0909, with resistance to gentamicin was *R=*-0.1355 with CI of − 0.5887 to 0.3825, *P=*0.0769, with resistance to levofloxacin was *R=*-0.06091 with CI of − 0.5373 to 0.4449, *P*=0.1291, with resistance to meropenem was *R=*-0.3035 with CI of − 0.7404 to 0.3138, *P*=0.0256, with resistance to piperacillin was *R=*-0.2873 with CI of − 0.7323 to 0.3297, *P*=0.0350, with resistance to trimethoprim-sulfamethoxazole was *R=*-0.07902 with CI of − 0.5501 to 0.4302, *P*=0.1120, with resistance to tetracycline was *R=*-0.04882 with CI of − 0.5286 to 0.4546, *P*=0.1763, with resistance to ticarcillin-k clavulanate was *R=*-0.2982 with CI of − 0.7378 to 0.3190, *P=*0.0350, with resistance to tobramycin was *R=*0.02673 with CI of − 0.4719 to 0.5124, *P*=0.3280. These analyses support an inverse correlation that was significant between hemolysis and resistance to ticarcillin-k clavulanate, meropenem, and piperacillin.

Correlation analyses between *A. baumannii* strain motility and resistance to antibiotics was also performed (Supplemental Fig. 4). Spearman’s test of correlation between *A. baumannii* strain motility and resistance to ampicillin-sulbactam indicated *R=*-0.3258 with a 95% confidence interval (CI) of − 0.7051 to 0.1985, *P*=0.0430. Spearman’s correlation of biofilm formation with resistance to amikacin was *R=*-0.01188 with CI of − 0.5014 to 0.4834, *P*=0.2955, with resistance to ceftriaxone or ceftoxamine was *R=*-0.2764 with CI of − 0.6767 to 0.2502, *P*=0.0362, with resistance to ceftazidime or ciprofloxacin was *R=*-0.2897 with CI of − 0.46845 to 0.2365, *P*=0.0284, with resistance to cefepime was *R=*-0.1318 with CI of − 0.5862 to 0.3857, *P*=0.1787, with resistance to gentamicin was *R=*-0.2390 with CI of − 0.6544 to 0.2873, *P*=0.0676, with resistance to levofloxacin was *R=*-0.3238 with CI of − 0.47040 to 0.2007, *P*=0.0311, with resistance to meropenem was *R=*0.1938 with CI of − 0.4152 to 0.6828, *P*=0.2583, with resistance to piperacillin was *R=*0.1789 with CI of − 0.4278 to 0.6745, *P*=0.2909, with resistance to trimethoprim-sulfamethoxazole was *R=*-0.09783 with CI of − 0.5632 to 0.4146, *P*=0.1626, with resistance to tetracycline was *R=*-0.1013 with CI of − 0.5656 to 0.4117, *P*=0.2342, with resistance to ticarcillin-k clavulanate was *R=*0.1155 with CI of − 0.4793 to 0.6376, *P*=0.3577, with resistance to tobramycin was *R=*=0.03102 with CI of − 0.35156 to 0.4686, *P*=0.2851. These data indicate an inverse correlation that was significant between motility and resistance to ampicillin-sulbactam, ceftriaxone, ceftoxamine, ceftazidime, ciprofloxacin, or levofloxacin.

Spearman’s test of correlation between *A. baumannii* strain biofilm formation and resistance to antibiotics was performed (Supplemental Fig. 5). Correlation between biofilm quantification and ampicillin-sulbactam resistance indicated *R=*0.1117 with a 95% confidence interval (CI) of − 0.4029 to 0.5727, *P*=0.3338. Spearman’s correlation of biofilm formation with resistance to amikacin indicated *R=*-0.2008 with CI of − 06309 to 0.3236, *P*=0.0966, with resistance to ceftriaxone or ceftoxamine indicated *R=*-0.008752 with CI of − 0.4991 to 0.4858, *P*=0.2419, with resistance to ceftazidime or ciprofloxacin indicated *R=*0.05025 with CI of − 0.4534 to 0.5296, *P*=0.2954, with resistance to cefepime indicated *R=*0.1690 with CI of − 0.3529 to 0.6106, *P*=0.2559, with resistance to gentamicin indicated *R=*0.1918 with CI of − 0.3320 to 0.6252, *P*=0.2287, with resistance to levofloxacin indicated *R=*0.004231 with CI of − 0.4893 to 0.4957, *P*=0.2974, with resistance to meropenem indicated *R=*0.1754 with CI of − 0.4308 to 0.6726, *P*=0.2811, with resistance to piperacillin indicated *R=*0.08909 with CI of − 0.4996 to 0.6215, *P*=0.3552, with resistance to trimethoprim-sulfamethoxazole indicated *R=*0 with CI of − 0.4925 to 0.4925, *P*=0.2681, with resistance to tetracycline indicated *R=*-0.3644 with CI of − 0.7265 to 0.1561, *P*=0.0368, with resistance to ticarcillin-k clavulanate indicated *R=*0.1083 with CI of − 0.4849 to 0.6332, *P*=0.3661, with resistance to tobramycin indicated *R=*0.1733 with CI of − 0.3490 to 0.6134, *P*=0.2576. Together, these results demonstrate that high biofilm formation was correlated with increased susceptibility to tetracycline, but not with any other antimicrobial resistance patterns detected.

## Discussion

Strains of *A. baumannii* were selected from diverse anatomical and disease origin for phenotypic analyses. We chose to study antimicrobial susceptibility in tandem with important virulence factors such as biofilm formation, motility, and hemolysis induction, because these processes have been implicated as critical for colonization and invasion of the vertebrate host (6), and we hypothesized that there could be correlations between the phenotypes of virulence, anatomical origin, and antimicrobial susceptibility. Of the 17 clinical *A. baumannii* strains evaluated, 11 (64.7%) were resistant to three or more classes of antibiotics and thus qualified to be designated as multi-drug resistant (MDR). Approximately 35.3% of strains were non-susceptible to at least one agent in all but two or fewer antimicrobial categories and qualified to be designated as extremely drug resistant (XDR). Additionally, 17.6% of *A. baumannii* strains in the cohort of clinical isolates from Nashville, Tennessee exhibited at least intermediate resistance to all antibiotics tested, and were qualified to be designated as pan-drug resistant (PDR). In comparison, a recent survey of *A. baumannii* isolates from ICU patients revealed 100% of isolates qualified as MDR and 32% as XDR [19]. A previous study from 2018 of antimicrobial susceptibility of 621 carbapenem-nonsusceptible *A. baumannii* isolates reported by the Emerging Infections Program Sites (which include data collection from Nashville, Tennessee) from 2012 to 2015 indicate that, among surveyed clinical isolates, 56.9% were susceptible to tobramycin, 61.1% were susceptible to amikacin, 30.7% were susceptible to gentamicin, 3.5% were susceptible to levofloxacin, 1.9% were susceptible to ciprofloxacin, 16.1% were susceptible to ceftazidime, 12.1% were susceptible to cefepime, 3.5% were susceptible to piperacillin/tazobactam, 36.1% were susceptible to ampicillin/sulbactam, and 17.2% were susceptible to trimethoprim/sulfamethoxazole [22]. In our study, among the 17 clinical strains surveyed, 47.1% were susceptible to tobramycin, 58.8% were susceptible to amikacin, 35.3% were susceptible to gentamicin, 35.3% were susceptible to levofloxacin, 35.3% were susceptible to ciprofloxacin, 35.3% were susceptible to ceftazidime, 35.3% were susceptible to cefepime, 17.6% were susceptible to piperacillin/tazobactam, 52.9% were susceptible to ampicillin/sulbactam, and 35.3% were susceptible to trimethoprim/sulfamethoxazole. Together, these results indicate that our small pilot sample of *A. baumannii* strains exhibited similar antimicrobial susceptibility patterns to previously published studies with respect to tobramycin, amikacin, cefepime, and gentamicin, but were slightly more susceptible to levofloxacin, ciprofloxacin, ceftazidime, piperacillin, ampicillin/sulbactam, and trimethoprim/sulfamethoxazole.

This sample of strains was further characterized to determine potential virulence phenotypes that could be associated with anatomical site of isolation, antimicrobial susceptibility patterns, or other features. Our results demonstrated that wound-isolated strains were significantly more motile than strains isolated from blood, urine or Foley catheter, or sputum/bronchial wash. Analysis of biofilm formation also revealed that isolates derived from patient blood samples formed significantly more biofilm than isolates from sputum or bronchial wash, or isolates from wound samples. Both biofilm and motility phenotypes have been shown to vary widely across clinically isolated strains of *A. baumannii* [23] and both processes have both been shown to be regulated by overlapping signaling networks in this pathogen [24, 25]. Although biofilm and motility phenotypes varied widely across strains in our study as well, we observed an inverse relationship between motility and biofilm formation in the 17 clinical isolates examined. Indeed, a similar relationship has been observed between biofilm and swarming motility in other gram negative ESKAPE (*Escherichia coli*, *Staphylococcus aureus*, *Klebsiella pneumoniae*, *Acinetobacter baumannii*, *Pseudomonas aeruginosa*, and *Enterococcus faecalis*) pathogens, such as *Pseudomonas aeruginosa* [26], indicating environmental signals, genetic elements, and other factors could influence the shift from motility to sessile biofilm formation across genera.

Motility in *A. baumanni* was also inversely correlated with hemolysis. Although the molecular mechanism by which this regulation occurs remains obscure, co-regulation of stress response, motility, biofilm formation, and hemolysis has been demonstrated in other bacterial pathogens, such as *Vibrio alginolyticus* [27]. In this pathogen, alternate sigma factor RpoX is implicated as a critical regulator of the expression of virulence factors associated with these important processes. It remains possible that a repertoire of regulators including sigma factors, could be important for governing the inverse relationships seen with these phenotypes.

In addition to the inverse relationship observed between motility and hemolysis, our analyses support an inverse correlation that was significant between hemolysis and resistance to several antibiotics as well as an inverse correlation that was significant between motility and antibiotic resistance. Motility and antibiotic resistance have been linked in a variety of pathogens such as *Campylobacter jejuni,* in which enhanced motility is associated with increased resistance to polymyxin B and ciprofloxacin [28]. In *P. aeruginosa*, quorum sensing inhibition has been shown to inhibit motility, biofilm formation, and resistance to meropenem [29]. Thus, it is plausible to hypothesize that these phenotypes are governed by similar overlapping regulatory networks in *A. baumannii*.

### Limitations of this study

Our study has several limitations. First, our sample size of strains was low due to the nature of this pilot study. Second, we did not have minimum inhibitory concentration (MIC) values for the antimicrobial activity, and although Clinical and Laboratory Standards Institute (CLSI) methods were applied by the participating clinical laboratory, some margin of error could be introduced due to local discrepancies. In future studies, the number of samples will be expanded, and MIC values will be determined to enhance the statistical power and refinement of this pilot study.

## Supplementary Information

**Additional file 1: Supplemental Figure 1.** Analysis of hemolysis phenotypes from *A. baumannii* laboratory strains isolated patients in Nashville, Tennessee. Blood agar plates 24 h post-inoculation with *A. baumannii* clinical isolate strains. Qualitative analysis of bacterial hemolysis as determined by diameter and intensity of bacterial lysis of sheep blood cells present on the plate.

**Additional file 2: Supplemental Figure 2.** Analysis of motility and biofilm phenotypes from *A. baumannii* laboratory strains 19,606 T and 17,978. A) Motility agar plates 24 h post-inoculation with *A. baumannii* clinical isolate strains. B) Crystal violet stained *A. baumannii* biofilms in polystyrene tubes. C) Quantitative analysis of bacterial motility as determined by measurement of diameter of bacterial cells present on the plate. Bars indicate mean values (+/− standard error mean error bars) with individual biological replicates indicated by discrete points (*n*=4). D) Quantitative analysis of ratio of biofilm to biomass. Biofilm was determined by solubilization of crystal violet and spectrophotometric measurement at OD_560_. Biomass was determined by spectrophotometric measurement at OD_600_. Bars indicate mean values (+/− standard error mean error bars) with individual data points indicated by discrete points (*n*=3 biological replicates with 3–4 technical replicates per experiment).

**Additional file 3: Supplemental Figure 3.** Analysis of the correlation between hemolysis and antimicrobial susceptibility phenotypes from *A. baumannii* strains isolated patients in Nashville, Tennessee. Qualitative analysis of hemolysis was scored as 1= low, 2= moderate, 3= high. Antimicrobial susceptibility was scored as 0= susceptible, 1= intermediate, 2= non-susceptible. Spearman’s correlation analyses were performed for each strain to determine the relationship between hemolysis and antimicrobial susceptibility.

**Additional file 4: Supplemental Figure 4.** Analysis of the correlation between motility and antimicrobial susceptibility phenotypes from *A. baumannii* strains isolated patients in Nashville, Tennessee. Quantitative analysis of bacterial motility as determined by measurement of diameter of bacterial cells present on the plate. Antimicrobial susceptibility was scored as 0= susceptible, 1= intermediate, 2= non-susceptible. Spearman’s correlation analyses were performed for each strain to determine the relationship between motility and antimicrobial susceptibility.

**Additional file 5: Supplemental Figure 5.** Analysis of the correlation between biofilm and antimicrobial susceptibility phenotypes from *A. baumannii* strains isolated patients in Nashville, Tennessee. Biofilm was determined by solubilization of crystal violet and spectrophotometric measurement at OD_560_. Biomass was determined by spectrophotometric measurement at OD_600_. Antimicrobial susceptibility was scored as 0= susceptible, 1= intermediate, 2= non-susceptible. Spearman’s correlation analyses were performed for each strain to determine the relationship between biofilm and antimicrobial susceptibility.

## Data Availability

The datasets used and/or analysed during the current study available from the corresponding authors upon reasonable request.

## Abbreviations

DNA: Deoxyribonucleic acid
ICU: Intensive care unit
VAP: Ventilator-acquired/associated pneumonia
AHLs: Acyl homoserine lactones
ISO: International Organization for Standardization
A/S: Ampicillin-sulbactam
AK: Amikacin
CAX: Ceftriaxone
CAZ: Ceftazidime
CFT: Cefotaxime
CP: Ciprofloxacin
CPE: Cefepime
GM: Gentamicin
LVX: Levofloxacin
MER: Meropenem
PI: Piperacillin
T/S: Trimethoprim-sulfamethoxazole
TEq: Tetracycline
TIM: Ticarcillin-K clavulanate
TO: Tobramycin
IRB: Institutional Review Board
LB: Luria-Bertani
MDR: Multi-drug resistant
CI: Confidence interval
XDR: Extremely drug resistant
ESKAPE: *E**scherichia coli*, *S**taphylococcus aureus*, *K**lebsiella pneumoniae*, *A**cinetobacter baumannii*, *P**seudomonas aeruginosa*, and *E**nterococcus faecalis*
MIC: Minimum inhibitory concentration
CLSI: Clinical and Laboratory Standards Institute
