# Supplementary figures and images for: Analysis of virulence phenotypes and antibiotic resistance in clinical strains of Acinetobacter baumannii isolated in Nashville, Tennessee
Source: BMC Microbiol. 2021 Jan 9;21:21. doi: 10.1186/s12866-020-02082-1 (PMC7796680; doi:10.1186/s12866-020-02082-1)

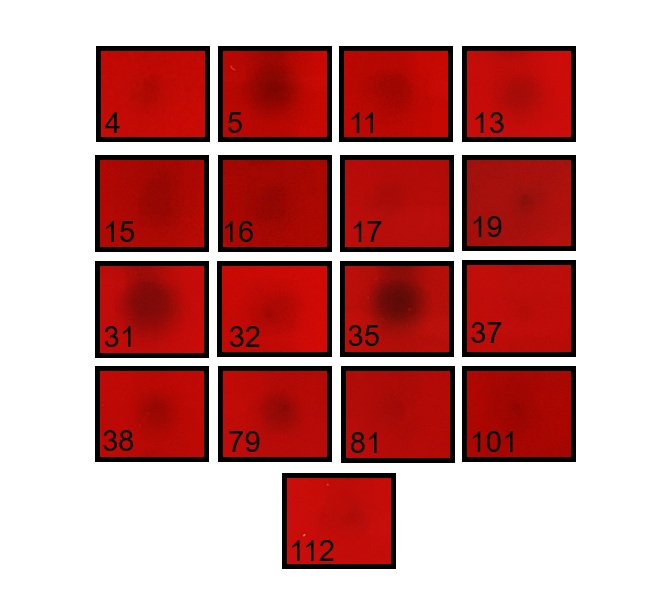

Supplement: Supplementary file 1 — Additional file 1: Supplemental Figure 1. Analysis of hemolysis phenotypes from A. baumannii laboratory strains isolated patients in Nashville, Tennessee. Blood agar plates 24 h post-inoculation with A. baumannii clinical isolate strains. Qualitative analysis of bacterial hemolysis as determined by diameter and intensity of bacterial lysis of sheep blood cells present on the plate. [file 12866_2020_2082_MOESM1_ESM.tif]

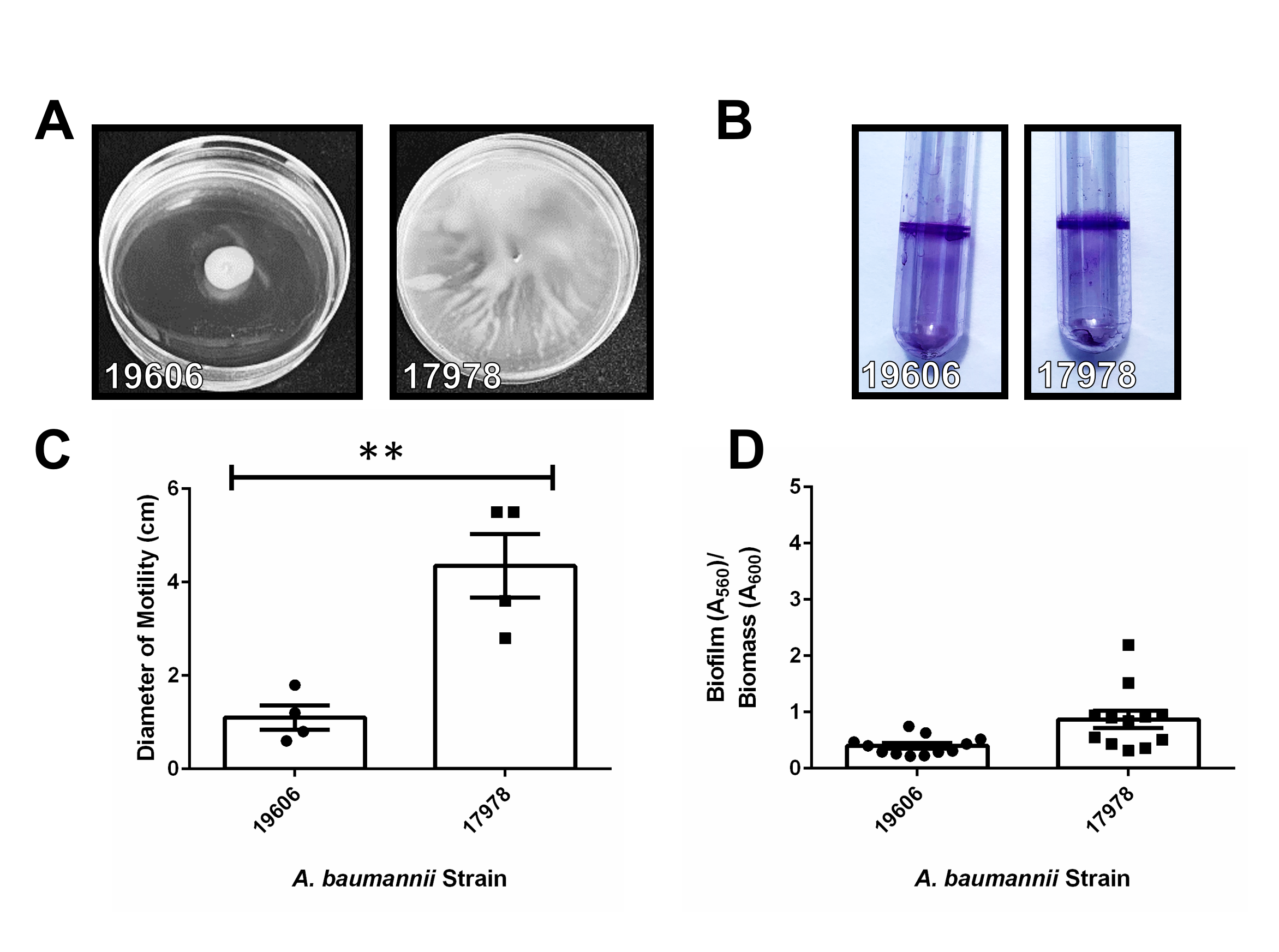

Supplement: Supplementary file 2 — Additional file 2: Supplemental Figure 2. Analysis of motility and biofilm phenotypes from A. baumannii laboratory strains 19,606 T and 17,978. A) Motility agar plates 24 h post-inoculation with A. baumannii clinical isolate strains. B) Crystal violet stained A. baumannii biofilms in polystyrene tubes. C) Quantitative analysis of bacterial motility as determined by measurement of diameter of bacterial cells present on the plate. Bars indicate mean values (+/− standard error mean error bars) with individual biological replicates indicated by discrete points (n=4). D) Quantitative analysis of ratio of biofilm to biomass. Biofilm was determined by solubilization of crystal violet and spectrophotometric measurement at OD560. Biomass was determined by spectrophotometric measurement at OD600. Bars indicate mean values (+/− standard error mean error bars) with individual data points indicated by discrete points (n=3 biological replicates with 3–4 technical replicates per experiment). [file 12866_2020_2082_MOESM2_ESM.tif]
